# Supplementary material for: Dynamic Interplay Between miR-124-3p and EGF in the Regulation of Overgrowth via RNA Signaling
Source: Biomolecules. 2025 Aug 18;15(8):1186. doi: 10.3390/biom15081186 (PMC12384290; doi:10.3390/biom15081186)
Supplement: Supplementary file 1 [file biomolecules-15-01186-s001.zip › biomolecules-3762685-supplementary.pdf]

Supplemental Materials

Table S1. List of genes used in mRNA expression by qPCR studies and access information

Genes	Probe Assay ID
Sox8	Mm.PT.58.8013006
Sox9	Mm.PT.58.42739087
Sox10	Mm.PT.58.42371609
Dcx	Mm.PT.58.28380868
Neurod1	Mm.PT.58.12535284
Dmrta1	Mm.PT.58.41362284
Ptbp1	Mm.PT.58.14137568
Ptbp2	Mm.PT.58.6831641
Ctdsp1	Mm.PT.58.41127385
Ghrh	Mm.PT.58.28988755
Gh	Mm.PT.58.41753888.gs
Ghr	Mm.PT.58.12306268
Igf1	Mm.PT.58.5811533
Igf1r	Mm.PT.58.11619137
Igfbp1	Mm.PT.58.45852897
Igfbp5	Mm.PT.58.11593699
Actb	Mm.PT.39a.22214843.g

Table S2. The sequence of miRNA used for microinjection technique and list of miRNAs used for miRNA expression by qPCR and access information

Gene	Accession number	Sequense
miR-124-3p (RNA for microinjection)	MIMAT 0000134	UAAGGCACGCGGUGAAUGCC
miR-124-3p (Primer for qPCR)	GeneGlobe ID - YP02119832	
miR-124-5p (Primer for qPCR)	GeneGlobe ID - YP00204266	
U6 SnRNA	GeneGlobe ID - YP02119464	

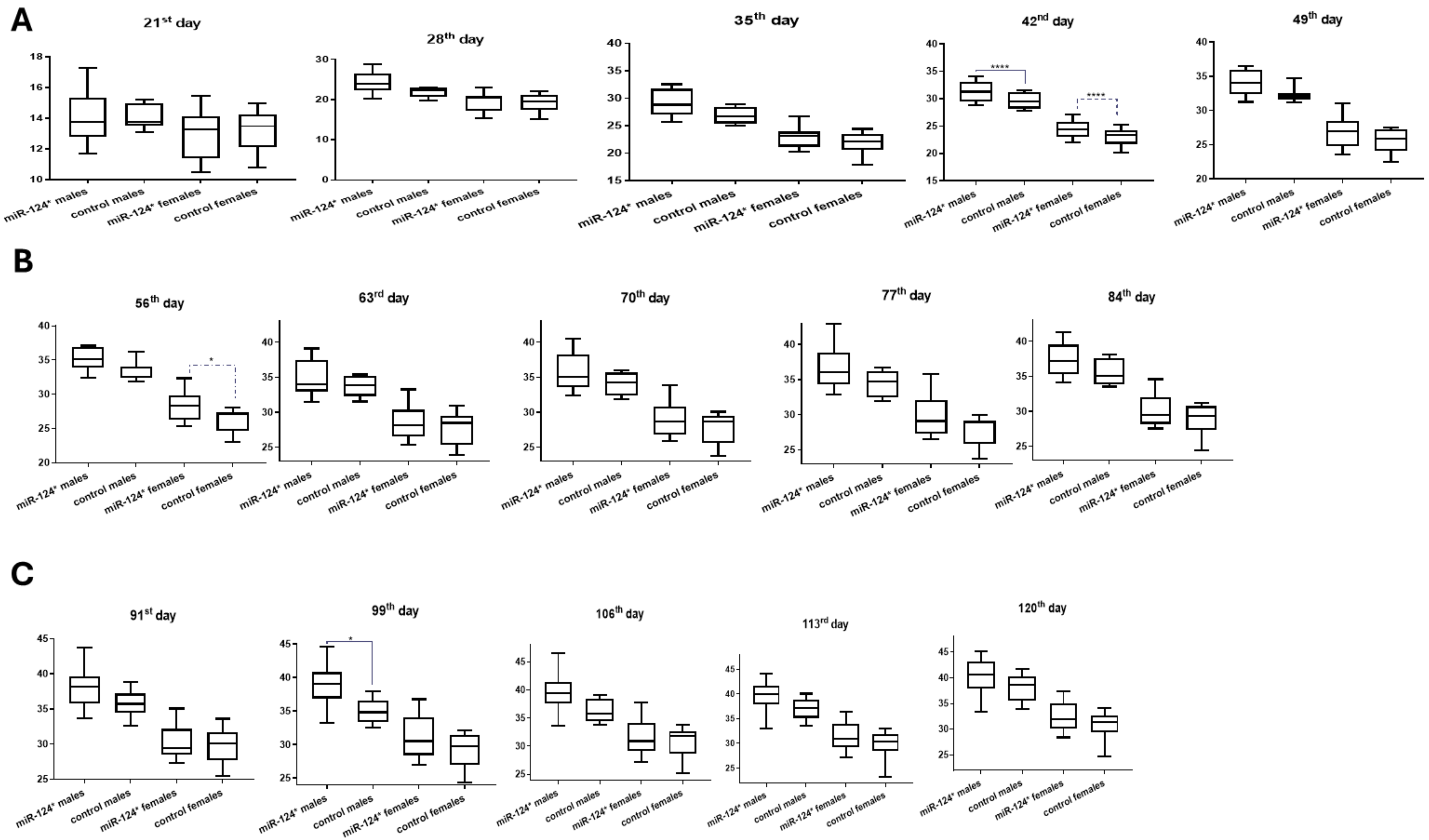

Figure S1: Weight graphs were obtained by weighing the mice weekly for 120 days after sex separation (day 21).

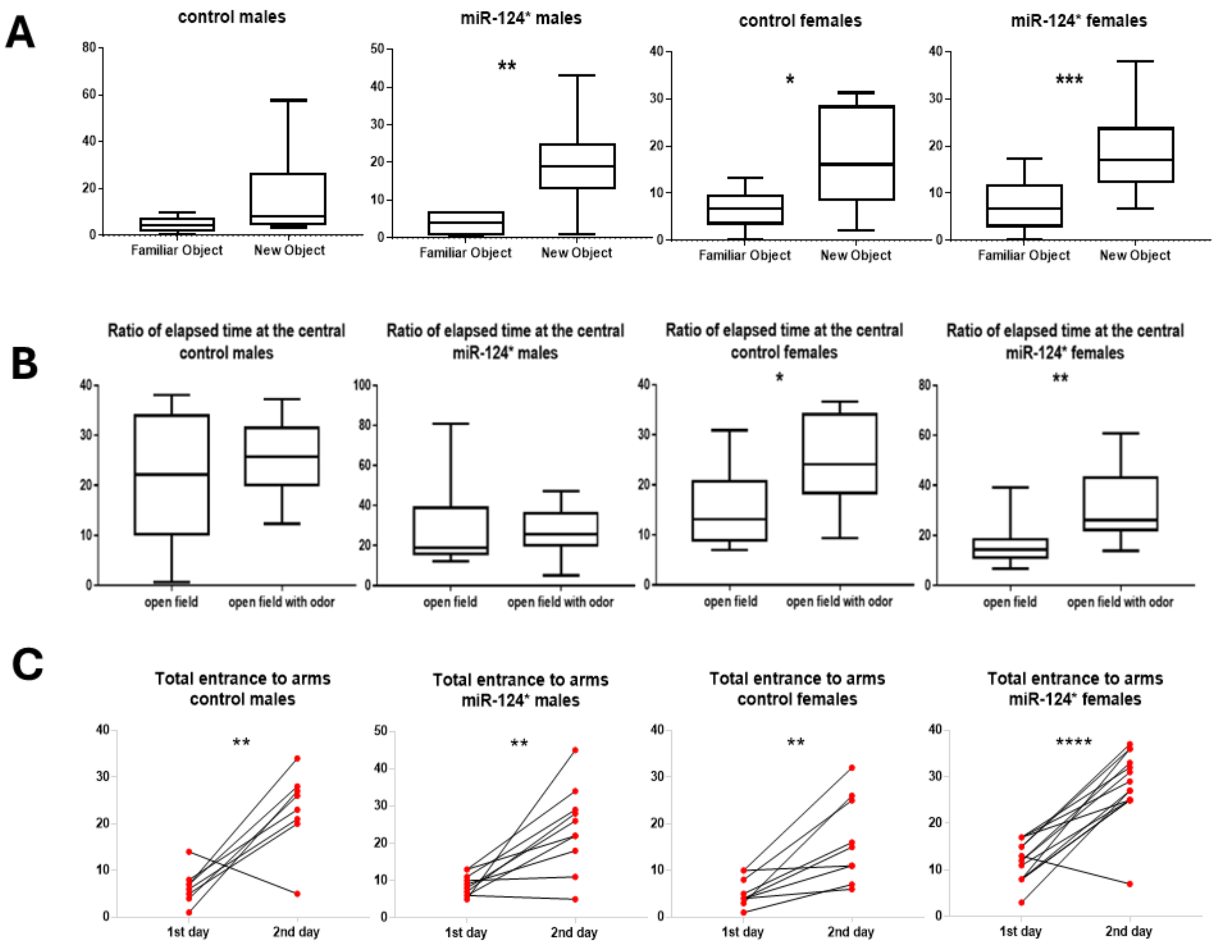

Figure S2: A) Novel Object; comparison of total exploration time between new object and familiar object, **B)** Odour test/ open field ratio; data comparing time spent in the center with and without a cinnamon stick placed in the open field arena, reflecting group differences, **C)** Y maze; Comparison of total entrance to arms across groups between Day 1 and Day 2. *p < 0.05, **p<0,01, ***p < 0.001, ****p < 0.0001

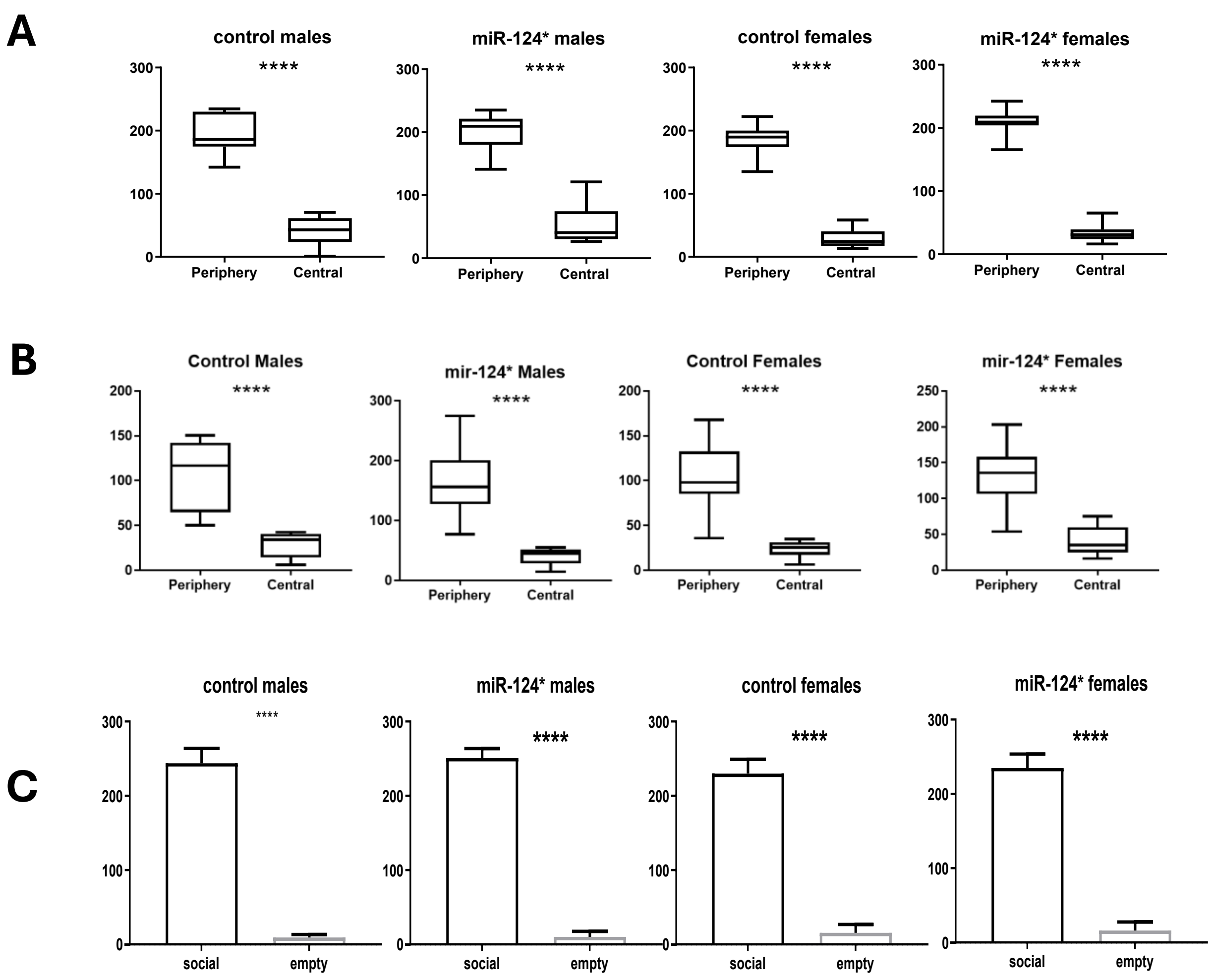

Figure S3: A) Comparison of total time spent in the center and periphery across groups in the open field test, **B)** Comparison of total time spent in the center and periphery across groups after introducing a cinnamon stick to the center of the open field arena, **C)** Comparison of time spent in the "social" versus "empty" chamber across groups in the social interaction test. *p < 0.05, **p<0,01, ***p < 0.001, ****p < 0.0001.

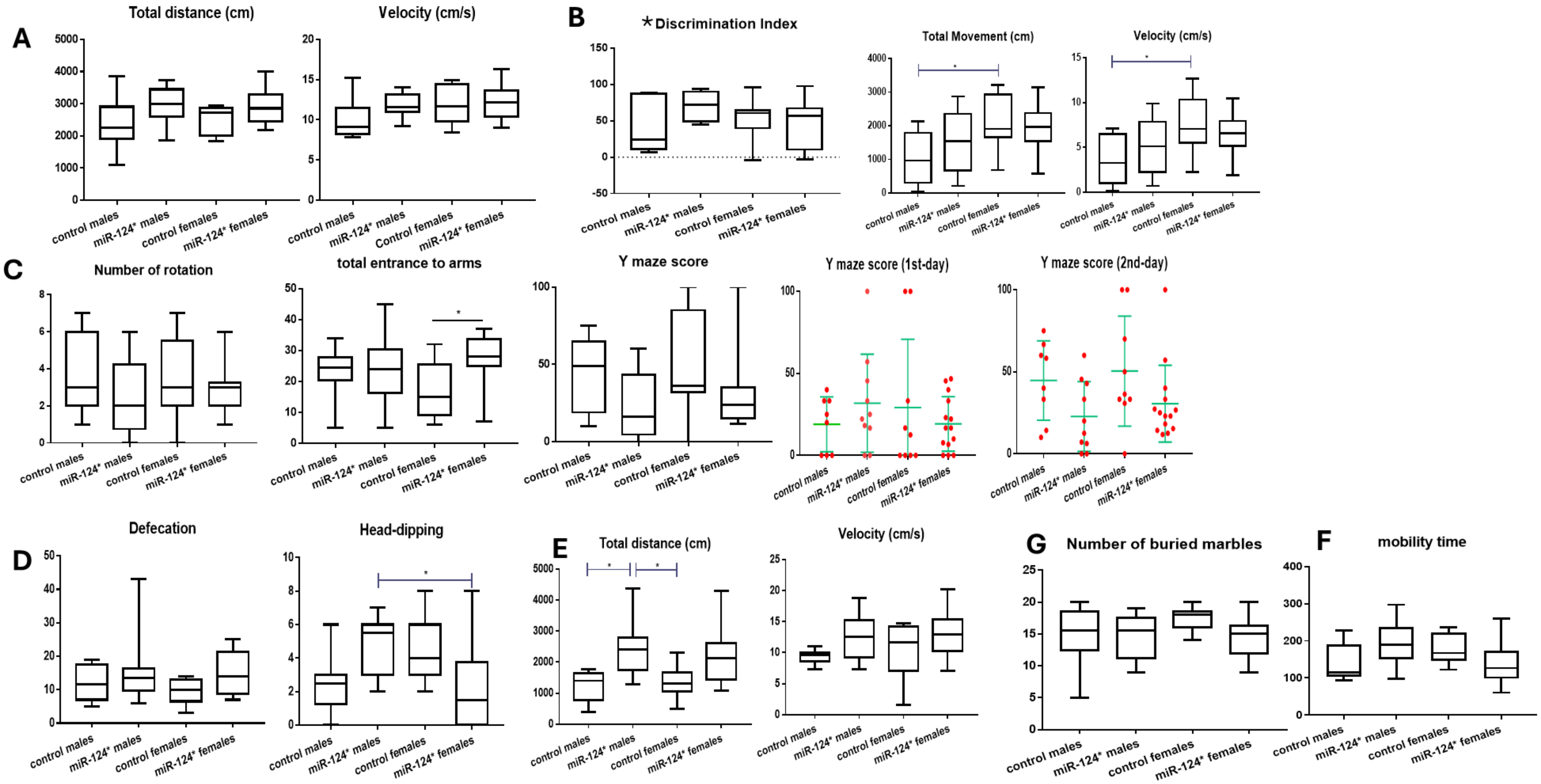

Figure S4: A) Open field; total distance and velocity comparison between groups, **B)** Novel Object; discrimination index (*), total distance and velocity comparison between groups, **C)** Y Maze; Comparison of the number of rotations, arm entries, Y-maze scores across groups based on Day 2 data, and Y maze scores across groups between Day 1 and Day 2. **D)** Hole Board; comparison of the number of defecations and head-dipping between groups, **E)** Odour Test; total distance and velocity comparison between groups, **G)** Marble Test; comparison of buried marble number, **F)** Tail Suspension; comparison of mobility time between groups. * Discrimination index: exhibits the percentage of the total time the mice spend with the new object. *$p < 0.05$, **$p<0,01$, ***$p < 0.001$, ****$p < 0.0001$.
